# The Effect of Adult Smoking Behavior on Children’s Exposure to Secondhand Smoke. An Analysis Based on Salivary Cotinine Levels Among Children in Dhaka and Karachi

**DOI:** 10.1093/ntr/ntae130

**Published:** 2024-06-19

**Authors:** Kamran Siddiqi, Charlie Welch, Rumana Huque, Romania Iqbal, Mona Kanaan, Masuma Pervin Mishu, Mariam Ahmad Khokhar, Sean Semple, CLASS III Trial Team, Aziz Sheikh, Catherine Hewitt, Catherine Jackson, Ian Kellar, Steve Parrott

**Affiliations:** Department of Health Sciences, University of York, York, UK; Hull York Medical School, University of York, York, UK; Department of Health Sciences, University of York, York, UK; ARK Foundation, Dhaka, Bangladesh; Department of Community Health Sciences, Aga Khan University, Karachi, Pakistan; Department of Health Sciences, University of York, York, UK; Department of Epidemiology and Public Health, University College London, London, UK; Department of Health Sciences, University of York, York, UK; Institute for Social Marketing and Health, University of Stirling, Stirling, UK

## Abstract

**Introduction:**

Exposure to secondhand smoke (SHS) risks children’s health. However, biomarkers are rarely used to study SHS exposure among children in low- and middle-income countries.

**Aims and Methods:**

We analyzed cross-sectional data collected between March and November 2022 for a cluster-randomized controlled trial investigating a Smoke-Free Intervention in 2769 children aged 9–15 in 74 schools (34 in Dhaka, Bangladesh, and 40 in Karachi, Pakistan). Children’s saliva was tested for the concentration of cotinine—a highly sensitive and specific biomarker for SHS exposure. Based on their reports, children’s homes were categorized as Nonsmoking Homes (NSH) when residents were nonsmokers; Smoke-free Homes (SFH) when residents and visitors smoked outdoors only; and Smoke-permitted Homes (SPH) when either residents or visitors smoked indoors. We compared cotinine concentrations across these home types and the two cities using a proportional odds model.

**Results:**

Overall, 95.7% of children (92% in Dhaka; and 99.4% in Karachi) had cotinine levels between 0.1 and 12 ng/mL, indicating SHS exposure. Median cotinine levels were higher in Karachi (0.58 ng/mL, IQR 0.37 to 0.93) than in Dhaka (0.27 ng/mL, IQR 0.16 to 0.49). Median cotinine concentration was also higher among children living in SPH than those in either NSH or SFH; with absolute differences of approximately 0.1–0.3 and 0.05 ng/mL, respectively.

**Conclusions:**

The level of SHS exposure in Dhaka and Karachi indicates widespread and unrestricted smoking. Smoking restrictions in households and enforcement of smoking bans are urgently needed.

**Implications:**

The high levels of SHS exposure in children living in SFH suggest parental behavior to hide their smoking and/or exposure in private vehicles or public spaces. It is important to advocate for SFH and cars to protect children from SHS exposure. However, these initiatives alone may not be enough. There is a need to enforce smoking bans in enclosed public places and transportation, as well as extend these bans to playgrounds, parks, fairgrounds, and other public spaces that children frequently visit. It is essential to complement smoking restrictions with tobacco cessation advice and support in these settings.

## Introduction

Exposure to secondhand smoke (SHS) is a significant health concern for children and adolescents worldwide.^1^ According to the Global Youth Tobacco Surveys conducted worldwide between 2010 and 2018, 63% of adolescents were exposed to SHS in the previous week, with 33% exposed daily.^2^ SHS exposure leads to lower respiratory tract infection^3^ and otitis media^4^ in children. Globally, SHS exposure is responsible for approximately 50 000 deaths and the loss of 4.5 million disability-adjusted life years per annum in children.^5^ Additionally, exposure to adult smoking at home^6^ and in public places^7^ is strongly associated with smoking among adolescents.

Over the past two decades, there has been a gradual decline in smoking prevalence globally.^8^ The overall proportion of children exposed to SHS has also fallen.^2^ However, this trajectory differs between high-income countries (HIC) and low- and middle-income countries (LMIC). HIC have witnessed much sharper declines in the proportion of children exposed to SHS, and the frequency/intensity of that exposure.^9,10^ In the United Kingdom, a decline of over 90% has occurred in nonsmokers’ exposure to SHS. Among children in England, the mean cotinine value (a highly sensitive and specific biomarker of SHS exposure)^11^ fell from 0.50 to 0.05 ng/mL between 1998 and 2018,^12^ and among nonsmoking adults in Scotland, it declined from 0.46 to 0.01 ng/mL between 1998 and 2016.^13^ Conversely, LMICs are experiencing a much slower decline, with an increase or no change in SHS exposure in public places in most countries.^2,14^ Despite being signatories to the World Health Organization’s Framework Convention on Tobacco Control (WHO FCTC), most LMIC countries are yet to offer comprehensive protection from SHS exposure. This indicates weak implementation of the FCTC and enforcement of smoking bans.^15^

It is crucial to prioritize efforts to protect children from SHS exposure in LMICs. To inform and evaluate smoke-free policy actions, there is an urgent need for robust data on SHS exposure and its determinants.

In this paper, we focus on Bangladesh and Pakistan—two populous countries with high tobacco burden. The adult prevalence of tobacco use and smoking is 19.1% and 12.4% in Pakistan^16^ and 35.3% and 18% in Bangladesh,^17^ respectively. Both countries have introduced laws prohibiting smoking in public places. However, Bangladesh’s smoke-free laws are comprehensively applied only in healthcare and education facilities. A few small-scale observational studies have reported poor compliance with smoke-free laws in both countries.^18,19^ We previously published an estimate of children’s exposure to SHS in Dhaka, Bangladesh using a low-cost particulate monitor (Dylos DC1700).^20^ However, it turned out to have lower specificity in Bangladesh than in HIC due to other sources of indoor air pollution and high levels of ambient air pollution and outdoor-to-indoor air exchange. Therefore, here we report the salivary cotinine levels measured in children in Dhaka, Bangladesh, and Karachi, Pakistan. Additionally, we assessed the association between indoor and outdoor adult smoking behaviors and salivary cotinine levels in children in both cities.

## Materials and Methods

### Study Design and Settings

We analyzed baseline (pre-intervention) data collected at the start of a cluster-randomized controlled trial of the Smoke-Free Intervention.^21^ The trial titled “Children Learning About Secondhand Smoke” (CLASS III) recruited 74 schools and 2769 children from Dhaka, Bangladesh and Karachi, Pakistan. We received ethics approvals from the University of York, the Bangladesh Medical Research Council, the Aga Khan University, and the Health Research Institute, Pakistan.

### Study Participants

A list of eligible schools (those that follow national curricula and have primary and secondary classes and smoke-free policies) was prepared in purposively selected localities. These were centrally located in some of the most densely populated neighborhoods of Dhaka and Karachi. From this list, 113 (50 in Dhaka and 63 in Karachi) schools were randomly selected and 74 (34 in Dhaka and 40 in Karachi) of them agreed to participate. Among these schools, 2769 children in year five (age range approximately 9–15 years old) were recruited after securing their assent and parental consent on an opt-out basis. Due to our primary interest in children exposed to SHS, we excluded children reporting active tobacco use.

### Data

We measured salivary cotinine among all enrolled children as a biomarker of SHS exposure. While nicotine has a very short half-life, cotinine, its proximate metabolite with a half-life of 17 hours, is detectable even 72 hours after SHS exposure.^11^ Once collected, saliva samples were stored at ambient temperature and sent to ACM Global Laboratories, UK (https://www.acmgloballab.com/) within 2 weeks. These were analyzed using a gas-liquid chromatography technique. Salivary cotinine was the primary outcome of the main trial.

The salivary cotinine concentrations were quantifiable between 0.1 and 50 ng/mL, denoted as “within limits of quantification” (WLQ) from hereafter. Less than 0.1ng/mL measurements were below the limit of quantification (BLQ), and those greater than 50 ng/mL were above the limit of quantification. In line with the planned analyses of the CLASS III trial, we use a cutoff of > 12 ng/mL^22^ to classify participants as potentially consuming smoked tobacco.

In addition, we also collected data on children’s sociodemographic variables, smoking-related behaviors including smoking restrictions at home, exposure to and visibility of tobacco smoke and their attitudes towards smoking.

### Data Collection

The data were collected during a classroom session. Participating children provided saliva samples in a sterile individually labeled tube and completed a questionnaire on a digital tablet. The questionnaire asked for outside space at the home (yes/no). For assessing smoking restrictions at home and exposure to and visibility of tobacco smoke, children were asked: Does anybody who lives with you smoke tobacco? (yes/no), Do people who live with you smoke? (anywhere inside your home/in some rooms in your home/only in one room in your home/only outside), Do people who live with you smoke in front of children? (yes/no), Do people who visit your home smoke? (anywhere inside your home/in some rooms in your home/only in one room in your home/only outside/nonsmoker visitors only), Do people who visit your home smoke in front of children? (yes/no), Did anyone smoke (in the last seven days) while you were in the vehicle (yes/no)? Have you been near someone smoking anywhere other than at home or in the vehicle in the past seven days? (yes/no).

Participants who indicated that nobody within their household smokes and that none of their household visitors smoke were categorized as being from nonsmoking homes (NSH). Participants who responded “Only outside” to both questions were categorized as being from Smoke-free Homes (SFH). All remaining combinations were categorized as Smoke-permitted Homes (SPH).^20^

### Data Analysis

The analyses focus on the participants with cotinine concentrations ≤12 ng/mL (including those with concentrations BLQ), hereafter referred to as the “main sample.” Key demographics of the main sample were summarized by home type (NSH, SFH, or SPH) and country (Bangladesh or Pakistan). Continuous/ordinal data (age and cotinine concentration) were summarized in terms of their arithmetic and geometric means, standard deviations, medians, and interquartile ranges. Categorical data were summarized in terms of frequencies and percentages.

For the main sample, we estimated the cumulative distribution functions of the cotinine measurements for each home type (NSH, SFH, or SPH) and country (Bangladesh or Pakistan) using a proportional odds model with fixed effects for home type, country, sex, age (modeled using a four-knot restricted cubic spline with knots placed at the 5th, 35th, 65th, and 95th percentiles of the observed age distribution), Outside space at the home (Yes/No) and SHS exposure outside of the home (Yes/No). For the latter indicator of SHS exposure outside the home, a value of “Yes” was assigned if the participant reported SHS exposure while they were in a vehicle, or if they reported SHS exposure anywhere other than at home or in a vehicle. Any participants that were missing values for any of the explanatory variables (home type, country, sex, age, and outside space at home or outside SHS exposure) were excluded. We used ordinal regression for two main reasons. Firstly, such an approach naturally handles the mixture of discrete and continuous outcome data that occur when using measurements of a biomarker that are subject to detection limits.^23^ Secondly, this approach facilitates inference regarding multiple aspects of the distribution of the outcome across participant characteristics using a single analysis model. For example, we can use a single model to compare and plot any quantiles of the distribution that are of interest (such as medians or upper or lower quartiles), or the probabilities of having measurements above or below any particular values of interest (such as the probability of having measurements that exceed 0.1 ng/mL).^23^ We used the fitted model to obtain and plot point estimates and 95% confidence intervals (based on the delta-method standard errors) for median cotinine concentrations by home type and country conditional on various representative values of the other fixed effects included in the model. We also used the fitted model to obtain and plot point estimates and 95% confidence intervals for the probability of having salivary cotinine concentration BLQ (ie, < 0.1 ng/mL) by home type and country conditional on various representative values of the other fixed effects included in the model. Analyses were conducted using Stata/MP v18.0^24^ and R version 4.2.3^25^ with the RMS package^26^ being used to fit the specified proportional odds model and obtain estimates of the quantities reported (medians and probabilities of having measurements BLQ).

## Results

**Figure 1.**
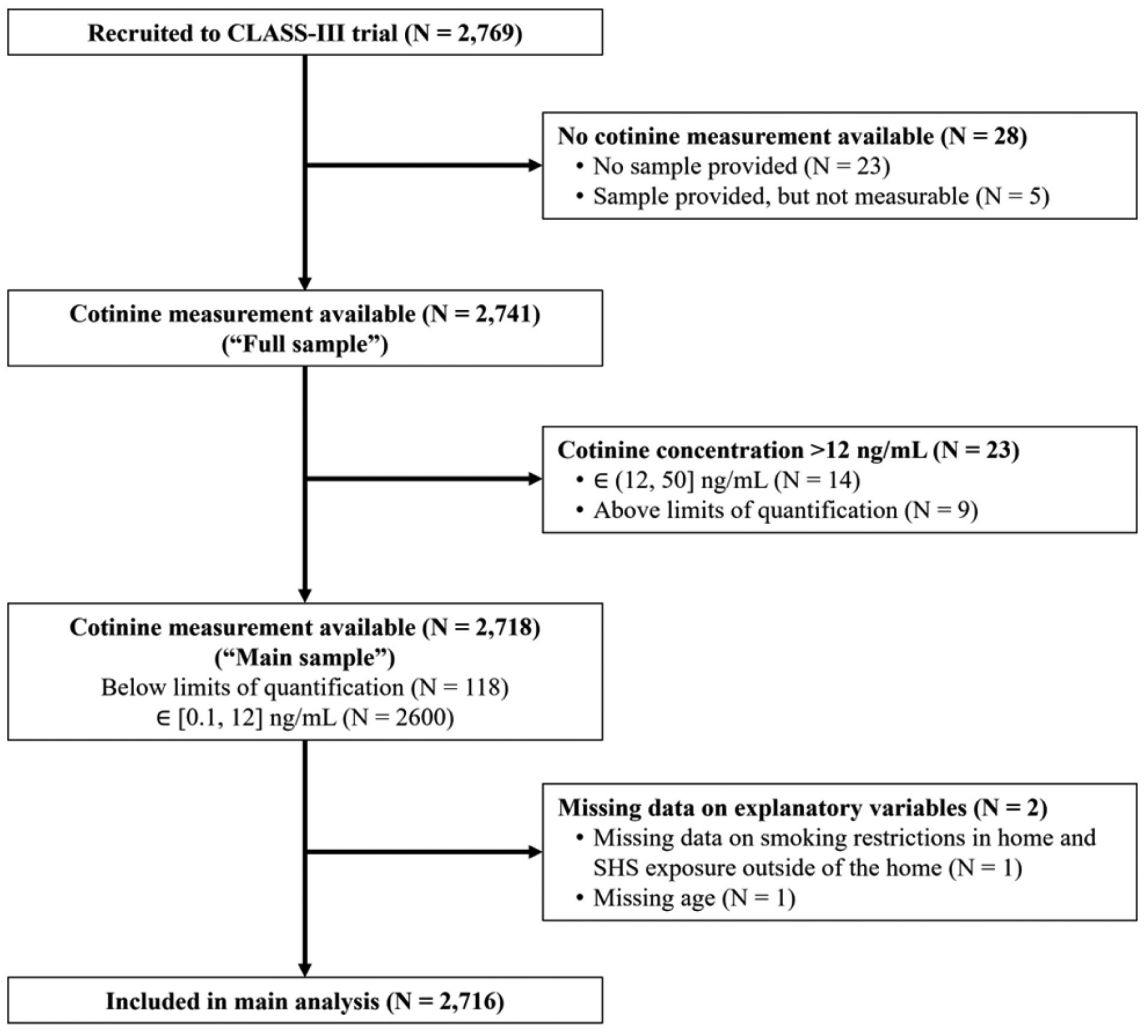
Schematic of data availability and analysis sets.

Saliva samples were collected from 2746 out of 2769 children recruited (Figure 1), although measurements could not be obtained from five (0.2%) samples due to insufficient volumes (*n* = 2) or problems reading the sample (*n* = 3). Of the 2741 children (1382 from Dhaka and 1359 from Karachi) with a valid measurement, 23 (0.8%) had concentrations > 12 ng/mL (14 were in the 12–50 ng/mL range and 9 were > 50 ng/mL, ie, above the limit of quantification). Hence there were 2718 children with measurements that were ≤ 12 ng/mL (the main sample), including 118 that were BLQ.

Table 1 shows that around 56% of children reported no smoking by household members and visitors (the NSH group). There was a fairly even split between SFH and SPH among the remaining children, with around 23% categorized as being from SFH and 21% being from SPH. Roughly half of the children in the NSH group, were from each country (49.7% Dhaka, 50.3% Karachi). However, this pattern was not replicated in either the SFH group (63% Dhaka) or the SPH group (62% Karachi). There was some evidence of an association between type of home and sex, with the proportion of males being lowest in the NSH category at around 43% and increasing to around 48% and 49% in the SFH and SPH categories, respectively. No such relationship was apparent concerning age, with the mean age being just over 11 years across all categories. Similarly, the proportion of participants reporting outside space at their homes was similar across the three categories (66%–68%). Around 60% of children reported SHS exposure outside of their home; 63% in SPH, 67% in SFH, and 53% in NSH. There was some evidence of an association between the type of home and cotinine concentration. Firstly, 95 (6.3%) children in NSH had BLQ cotinine concentrations compared to just 3% and 1% in SFH and SPH, respectively. Secondly, for all measures of central tendency reported, there is an apparent gradient, with children in NSH having the lowest measurements and those in SPH having the highest.

**Table 1.** Characteristics of the Main Sample by Home Type (Nonsmoking Homes, Smoke-Free Homes, or Smoke-Permitted Homes) and Overall. Numbers (Percentages) are Presented Unless Otherwise Specified

	NSH (*N* = 1517)	SFH (*N* = 618)	SPH (*N* = 582)	Total (*N* = 2717)
*Country, n (%)*				
Dhaka	754 (49.7)	390 (63.1)	224 (38.5)	1368 (50.3)
Karachi	763 (50.3)	228 (36.9)	358 (61.5)	1349 (49.7)
*Sex, n (%)*				
Male	648 (42.7)	296 (47.9)	282 (48.5)	1226 (45.1)
Female	869 (57.3)	322 (52.1)	300 (51.5)	1491 (54.9)
*Age (years)*				
N	1516	618	582	2716
Mean (SD)	11.3 (1.0)	11.3 (1.0)	11.4 (1.1)	11.3 (1.0)
Median (Q1, Q3)	11.2 (10.8, 12.0)	11.2 (10.8, 12.0)	11.2 (10.8, 12.0)	11.2 (10.8, 12.0)
Min, Max	8.0, 16.5	9.0, 15.3	8.0, 15.0	8.0, 16.5
*Outside space at home?, n (%)*				
No	489 (32.2)	197 (31.9)	199 (34.2)	885 (32.6)
Yes	1028 (67.8)	421 (68.1)	383 (65.8)	1832 (67.4)
*Reports SHS exposure outside of home?, n (%)*				
No	707 (46.6)	226 (36.6)	193 (33.2)	1126 (41.4)
Yes	810 (53.4)	392 (63.4)	389 (66.8)	1591 (58.6)
*Cotinine concentration (ng/mL)*				
Within [0.1, 12] ng/mL, *n* (%)	1422 (93.7)	600 (97.1)	577 (99.1)	2599 (95.7)
Arithmetic mean (SD)	0.57 (0.77)	0.67 (0.84)	0.80 (0.83)	0.64 (0.81)
Geometric mean (GSD)	0.40 (2.19)	0.46 (2.21)	0.58 (2.17)	0.45 (2.22)
Median** Q1, Q3)	0.35 (0.20, 0.63)	0.42 (0.26, 0.75)	0.57 (0.32, 0.97)	0.40 (0.23, 0.72)
BLQ (< 0.1 ng/mL), *n* (%)	95 (6.3)	18 (2.9)	5 (0.9)	118 (4.3)

*One of the 2718 participants in the main sample was missing data on smoking restrictions within the home and is therefore excluded from this table.

**This row provides the quartiles for the set of all measurements in the main sample (including those that were below the limit of quantification).

Table 2 shows that 1369 (50.4%) children were from Dhaka and 1349 (49.6%) from Karachi. The female-male ratio was approximately 55:45 across both countries and the mean/median ages were also similar. Around 65%–70% of participants reported having some outside space at their home and most participants (58.5%) reported SHS exposure outside their homes. As per Table 1, the proportion of children in NSH was similar across the two countries, but there were differences in the SFH and SPH, with Pakistani children being considerably more likely to report smoking indoors at their homes. The number and proportion of participants with salivary cotinine concentrations BLQ were considerably higher in Dhaka (110, 8.0%) as compared to Karachi (8, 0.6%). Likewise, all measures of central tendency show that measured cotinine concentrations were generally lower among Bangladeshi participants.

**Table 2.** Characteristics of the Main Sample by Country (Dhaka or Karachi) and Overall. Numbers (Percentages) are Presented Unless Otherwise Specified

	Dhaka (*N* = 1369)	Karachi (*N* = 1349)	Total (*N* = 2718)
*Type of home, n (%)*			
NSH	754 (55.1)	763 (56.6)	1517 (55.8)
SFH	390 (28.5)	228 (16.9)	618 (22.7)
SPH	224 (16.4)	358 (26.5)	582 (21.4)
Missing	1 (0.1)	0 (0.0)	1 (0.0)
*Sex, n (%)*			
Male	623 (45.5)	603 (44.7)	1226 (45.1)
Female	746 (54.5)	746 (55.3)	1492 (54.9)
*Age (years)*			
N	1369	1348	2717
Mean (SD)	11.24 (0.79)	11.43 (1.20)	11.33 (1.02)
Median (Q1, Q3)	11.20 (10.80, 11.87)	11.17 (10.75, 12.00)	11.19 (10.79, 12.00)
Min, Max	9.00, 14.75	8.00, 16.48	8.00, 16.48
*Outside space at home?, n (%)*			
No	415 (30.3)	470 (34.8)	885 (32.6)
Yes	954 (69.7)	879 (65.2)	1833 (67.4)
*Reports SHS exposure outside of home?, n (%)*			
No	592 (43.2)	534 (39.6)	1126 (41.4)
Yes	776 (56.7)	815 (60.4)	1591 (58.5)
Missing	1 (0.1)	0 (0.0)	1 (0.0)
*Cotinine concentration (ng/mL)*			
Within [0.1, 12] ng/mL, *n* (%)	1259 (92.0)	1341 (99.4)	2600 (95.7)
Arithmetic mean (SD)	0.46 (0.59)	0.82 (0.95)	0.65 (0.82)
Geometric mean (GSD)	0.32 (2.12)	0.60 (2.06)	0.45 (2.22)
Median^*^ (Q1, Q3)	0.27 (0.16, 0.49)	0.58 (0.37, 0.93)	0.40 (0.23, 0.72)
BLQ (< 0.1 ng/mL), *n* (%)	110 (8.0)	8 (0.6)	118 (4.3)

^*^This row provides the quartiles for the set of all measurements in the main sample (including those that were below the limit of quantification).

**Figure 2.**
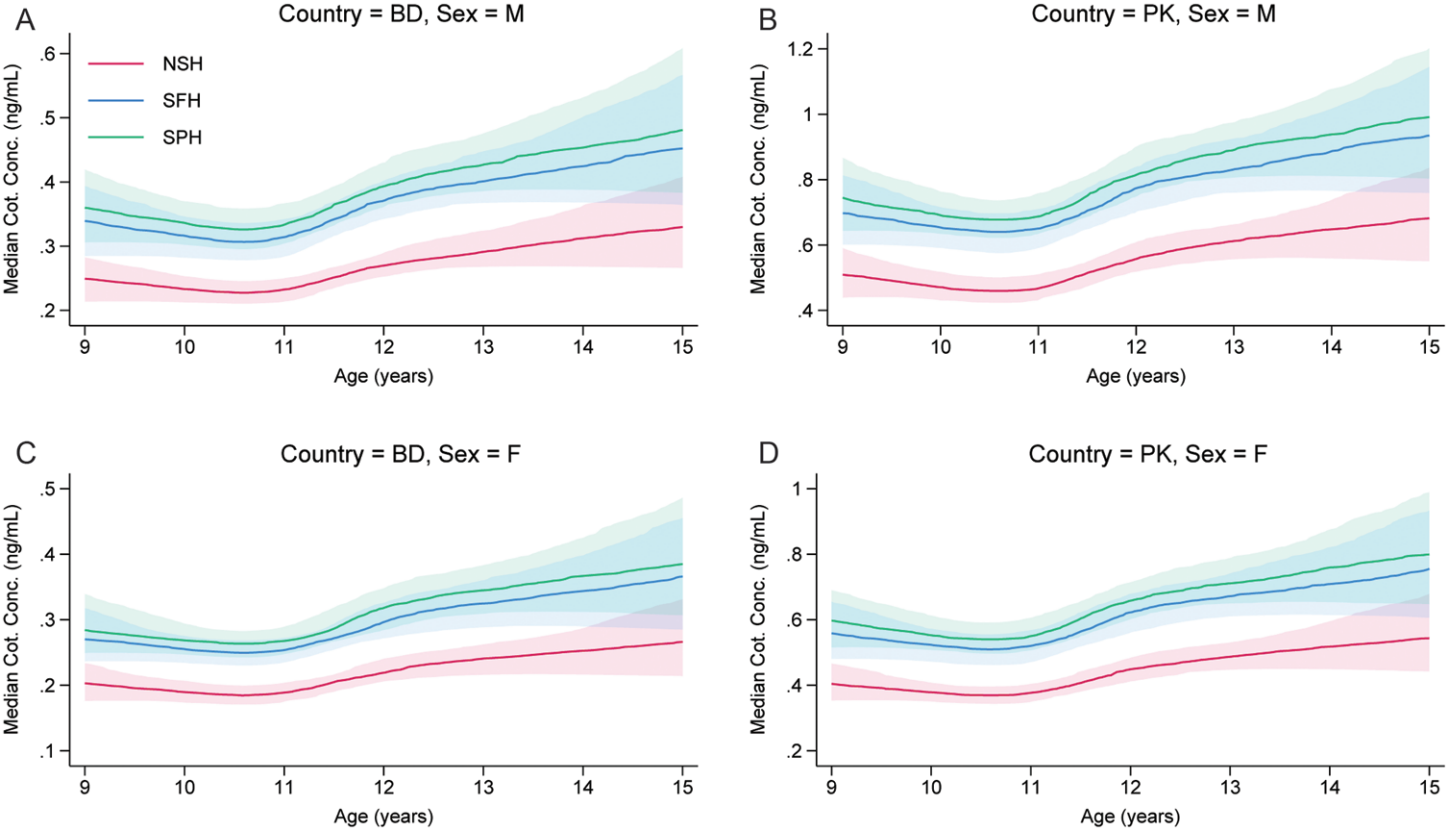
(A–D) Estimated median cotinine concentrations by type of home (NSH, Smoke-free Homes or Smoke-permitted Homes), conditional on age (9–15 years), country (Bangladesh = BD or Pakistan = PK), sex (Male = M or Female = F), outside space reported at home and SHS exposure reported outside of the home. The point estimates are shown as the darker line with the shaded areas showing pointwise 95% confidence intervals. Note the variation in the range of the *Y*-axis across the four plots.

Figure 2 suggests clear variation in median cotinine measurements across the three home types, with measurements from children in SPH generally being slightly higher than those from otherwise similar children resident in SFH, which in turn are generally higher than those in NSH. However, the absolute differences in medians were relatively small across all covariate patterns shown. Even when comparing children resident in NSHs to children in SPHs, the absolute differences in point estimates of the medians are generally around 0.1–0.3 ng/mL and are less than 0.05 ng/mL when comparing the SPH and SFH categories. Also evident is some variation in cotinine concentrations by age and sex, with children older than 11 years generally having slightly higher median measurements than children aged between 9 and 11, and males generally having slightly higher medians than females. Plots of the estimated probability of having a cotinine concentration BLQ by type of home conditional on various representative covariate patterns show a similar pattern, with children in NSH generally having higher probabilities of having measurements BLQ than children in the SFH and SPH categories (Appendix 1).

**Figure 3.**
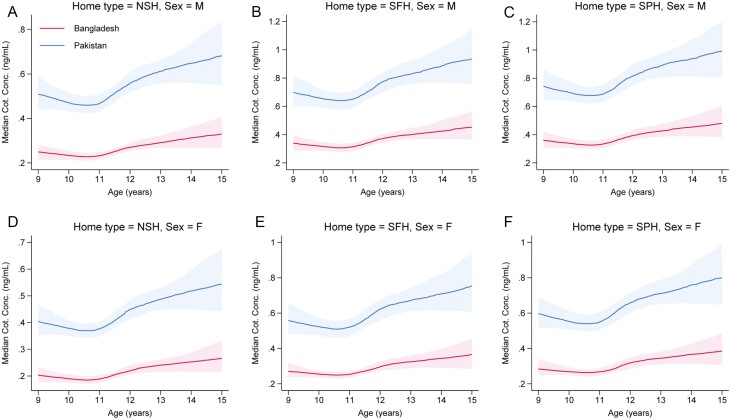
(A–F) Estimated median cotinine concentrations by country (Bangladesh = BD or Pakistan = PK), conditional on age (9–15 years), type of home (NSH, Smoke-free Homes, or Smoke-permitted Homes), sex (Male = M or Female = F), outside space reported at home and SHS exposure reported outside of the home. The point estimates are shown as the darker line with the shaded areas showing pointwise 95% confidence intervals. Note the variation in the range of the *Y*-axis across the four plots.

Figure 3 suggests some clear differences in median cotinine measurements across the two countries, with measurements from Pakistani participants generally being higher than those from otherwise similar Bangladeshi participants. Indeed, the point estimates of the medians for Pakistani children are approximately double those for otherwise similar Bangladeshi children. The absolute differences in medians are generally around 0.2–0.5 ng/mL depending on age, sex, and type of home. Plots of the estimated probability of having a cotinine concentration BLQ by country conditional on various representative covariate patterns show a similar pattern, with residency in Bangladesh being associated with a substantially higher probability of having a cotinine concentration BLQ (Appendix 1).

## Discussion

Among our sample of 2741 children, almost all (95.7%) were exposed to SHS. The estimates of the mean (arithmetic and geometric) and median cotinine concentration indicate a substantial level of SHS exposure in the two cities. This widespread SHS exposure is likely to be due to the high prevalence of male smoking in Bangladesh (33%)^27^ and Pakistan (21%),^16^ respectively. In the Global Adult Tobacco Surveys conducted in 21 countries, children’s exposure to SHS was directly linked to the rates of smoking in adults.^28^ Furthermore, the extent of SHS exposure indicates unrestricted smoking, both in private and public spaces.^29^ In our sample, children who were living with at least one smoker, 36.7% in Dhaka and 60.8% in Karachi reported indoor smoking at home. The difference in indoor smoking norms between the two countries could be the reason behind our finding of much higher salivary cotinine concentrations in children in Pakistan. In Pakistan, research related to indoor smoking is very limited, making it an under-researched area. According to Pakistan’s Demographic Health Survey, 40% of pregnant women reported being exposed to secondhand smoke (SHS) at home.^30^ Previous surveys of smoking restriction/permission at homes in Bangladesh, although limited in number and sample sizes, align with our findings.^14,31^

Ours is among a handful of studies conducted in LMICs assessing SHS exposure using exposure biomarkers. We conducted a previous, albeit smaller survey among schoolchildren in Dhaka in 2016 and observed similar findings.^14^ In another survey in Malaysia, 77% of school children had cotinine levels indicative of SHS exposure with a mean cotinine concentration of 0.4 6ng/mL.^32^ The mean cotinine estimates observed in our current study are also comparable to cotinine estimates observed over a generation ago in the United Kingdom; 0.50 ng/mL in England and 0.46 ng/mL in Scotland in 1998^12,13^ We also found that median cotinine levels of children in Karachi were around twice as high as those from otherwise similar children in Dhaka; this translates into an absolute difference in conditional medians of around 0.2–0.5 ng/mL. The smoking prevalence among males in Pakistan is less than in Bangladesh.^27,16^ Therefore, poorer adherence to smoking bans in public spaces and a significant lack of smoking restrictions in private spaces in Pakistan are likely to be responsible for the difference in SHS exposure between the two cities.

We also examined the association between the adult smoking behaviors of residents and visitors at homes and salivary cotinine levels in children in both cities. Compared to children living with nonsmokers, those living with adult smokers had higher levels of SHS exposure. Similarly, children living in houses permitting indoor smoking had slightly higher levels of SHS exposure than those where smoking was not permitted. The median cotinine concentration in the saliva of children living in homes permitting indoor smoking was around 0.1–0.3 ng/mL higher than those living in homes having no smokers or no permission to smoke indoors. However, the difference in median salivary cotinine concentration between those living in homes permitting indoor smoking and those restricting smoking to just outside spaces was considerably smaller, less than 0.05 ng/mL for all covariate patterns. Our data were gathered from children living in two of the most densely populated cities in the world, and therefore highly dense and/or multi-unit housing might have played a part in children’s exposure to tobacco use from nearby smoking-permitting homes. While two-thirds of homes had outdoor spaces, their proximity to indoor living spaces might not have completely protected children even if adults stepped outside to smoke.

The negative association between SHS exposure and smoking restrictions at home observed in our study has been found previously in several studies in LMICs.^33,34^ However, it is worth noting that biomarkers such as cotinine have been rarely utilized in LMICs^14,32^ as compared to HIC.^35–38^ A study conducted in the United Kingdom found that the cotinine levels were significantly lower in children whose parents did not smoke inside the house, even after adjusting for the average daily cigarette consumption of the parents.^36^ A more recent study conducted in the U.S. surveyed mothers regarding the smoking habits of their household. Also, the study evaluated child saliva samples for cotinine. The research concluded that children living in homes with smoking restrictions had a lower level of salivary cotinine and less risk of exposure to SHS compared to those living in homes with no restrictions.^39^ Like our study, these and other authors also noted that smoking restrictions may not be sufficient on their own to provide full protection to children from SHS exposure, especially those living with smokers.^40,41^ A combination of smoking restrictions at home and smoking cessation may be needed in this group.^42,43^ Furthermore, our study suggests that even living with nonsmokers may not be sufficient to provide full protection to children from SHS exposure. Those living in NSHs also had a substantial median cotinine value. If their self-reports on no smoking are accurate, this SHS exposure might have come from outside the homes. Therefore, both private and public indoor smoking restrictions may need to be implemented.^7^

Our study is one of the few to report on SHS exposure using exposure biomarkers among children in LMICs. However, there are some limitations to our findings. Firstly, the non-probability sampling strategy was used to recruit participants for the CLASS III trial means that our results cannot be generalized to the wider population of the two countries. However, the children were recruited from a mix of schools, both public and private, across a wide range of socioeconomic backgrounds in the two cities. Secondly, the variables used to capture indoor smoking behaviors were based on the children’s observations and their reporting accuracy, which were likely subject to observation and reporting biases, respectively. Parents may be good at hiding their smoking habits, especially indoor smoking, which could falsely characterize them as nonsmokers and their homes as smoke-free. The high concentration of cotinine found in children who reported living with nonsmokers or in smoke-free environments suggests that this may be the case. Finally, we were unable to accurately collect data on children’s exposure to smoking behaviors outside of their homes. This is due to the dynamic nature of such behaviors, making it difficult to report. However, new technologies have been developed to accurately record geospatial and temporal data, which could help address this gap in future studies. Given the high levels of cotinine found in children living in nonsmoking homes, accurately reporting their outside exposure is an important issue that needs to be addressed in future research.

Bangladesh and Pakistan are signatories to WHO FCTC and have introduced smoke-free laws in public places. While Pakistan’s legislation is comprehensive, Bangladesh’s smoke-free laws are only partial when applied to workplaces, restaurants, and public transport. Furthermore, previous research studies have expressed concerns about poor compliance with smoke-free laws in both countries.^18,19^ Exposure to SHS is a significant threat to the life chances of children in Bangladesh and Pakistan. Therefore, we call for a comprehensive approach to protect them from this harm. Enforcing smoking bans in public places, such as shops, restaurants, cafes, and public transport, should be given high priority. Smoking bans should also be extended to playgrounds, parks, fairgrounds, and other public spaces frequently visited by children. While advocating for smoke-free zones in private settings like homes and automobiles is crucial, this initiative alone might fall short of the comprehensive action required. Therefore, other evidence-based interventions should be delivered in a variety of healthcare and community settings, such as schools. Any of the above efforts should be complemented by offering tobacco cessation advice and support in these settings. Salivary cotinine levels could be used for benchmarking and setting targets for countries like Bangladesh and Pakistan. Measuring cotinine levels could supplement Global Youth Tobacco Surveys as part of the monitoring actions included in WHO’s MPOWER strategy.^44^

In summary, while SFH have the potential to greatly reduce children’s exposure to SHS, they may not entirely protect children from SHS exposure, especially in homes with nonsmokers. The effectiveness of smoking restrictions at home in fully protecting children from SHS exposure may depend on various factors, including the smoking behaviors of household members and the implementation of comprehensive tobacco control measures.

## Supplementary material

Supplementary material is available at *Nicotine and Tobacco Research* online.

ntae130_suppl_Supplementary_Appendix

## Data Availability

This data originates from a randomized controlled trial that is yet to publish its results. The data will be made publically available once the main trial findings have been published.

## References

[1] Alla F. Second-hand tobacco exposure in children: evidence for action. Lancet Public Health. 2021;6(8):e537–e538. 34274046 10.1016/S2468-2667(21)00128-6

[2] Ma C , Heiland EG, Li Z, et al. Global trends in the prevalence of secondhand smoke exposure among adolescents aged 12-16 years from 1999 to 2018: an analysis of repeated cross-sectional surveys. Lancet Glob Health. 2021;9(12):e1667–e1678. 34571047 10.1016/S2214-109X(21)00365-X

[3] Jones LL , Hashim A, McKeever T, et al. Parental and household smoking and the increased risk of bronchitis, bronchiolitis and other lower respiratory infections in infancy: systematic review and meta-analysis. Respir Res. 2011;12(1):5. 21219618 10.1186/1465-9921-12-5 PMC3022703

[4] Jones LL , Hassanien A, Cook DG, Britton J, Leonardi-Bee J. Parental smoking and the risk of middle ear disease in children: a systematic review and meta-analysis. Arch Pediatr Adolesc Med. 2012;166(1):18–27. 21893640 10.1001/archpediatrics.2011.158

[5] GBD Compare. Institute for Health Metrics and Evaluation. http://vizhub.healthdata.org/gbd-compare (Accessed October 31, 2023).

[6] Mays D , Gilman SE, Rende R, et al. Parental smoking exposure and adolescent smoking trajectories. Pediatrics. 2014;133(6):983–991. 24819567 10.1542/peds.2013-3003 PMC4035590

[7] Mishu MP , Siddiqui F, Shukla R, et al. The predictors of cigarette smoking, smokeless tobacco consumption and use of both forms in adolescents in South Asia: a secondary analysis of the Global Youth Tobacco Surveys (GYTS). Nicotine Tob Res. 2021;23(6):956–965. 33022045 10.1093/ntr/ntaa202

[8] Dai X , Gakidou E, Lopez AD. Evolution of the global smoking epidemic over the past half century: strengthening the evidence base for policy action. Tob Control. 2022;31(2):129–137. 35241576 10.1136/tobaccocontrol-2021-056535

[9] Merianos AL , Jandarov RA, Choi K, Mahabee-Gittens EM. Tobacco smoke exposure disparities persist in U.S. children: NHANES 1999-2014. Prev Med. 2019;123(1):138–142. 30902698 10.1016/j.ypmed.2019.03.028 PMC6534457

[10] Kuntz B , Lampert T. Social disparities in parental smoking and young children’s exposure to secondhand smoke at home: a time-trend analysis of repeated cross-sectional data from the German KiGGS study between 2003-2006 and 2009-2012. BMC Public Health. 2016;16(1):485. 27277721 10.1186/s12889-016-3175-x PMC4898452

[11] Benowitz NL , Bernert JT, Foulds J, et al. Biochemical verification of tobacco use and abstinence: 2019 update. Nicotine Tob Res. 2020;22(7):1086–1097. 31570931 10.1093/ntr/ntz132 PMC7882145

[12] Tattan-Birch H , Jarvis MJ. Children’s exposure to second-hand smoke 10 years on from smoke-free legislation in England: cotinine data from the Health Survey for England 1998-2018. Lancet Reg Health Eur. 2022;15(1):100315. 35146477 10.1016/j.lanepe.2022.100315 PMC8819129

[13] Semple S , Mueller W, Leyland AH, Gray L, Cherrie JW. Assessing progress in protecting non-smokers from secondhand smoke. Tob Control. 2019;28(6):692–695. 30158211 10.1136/tobaccocontrol-2018-054599 PMC6914375

[14] Shah S , Kanaan M, Huque R, et al. Secondhand smoke exposure in primary school children: a survey in Dhaka, Bangladesh. Nicotine Tob Res. 2017;21(4):416–423. 10.1093/ntr/ntx248 PMC6472694 29228385

[15] Semple S , Dobson R, O’Donnell R, et al. Smoke-free spaces: a decade of progress, a need for more? Tob Control. 2022;31(2):250–256. 35241597 10.1136/tobaccocontrol-2021-056556

[16] Saqib MAN , Rafique I, Qureshi H, et al. Burden of tobacco in pakistan: findings from global adult tobacco survey 2014. Nicotine Tob Res. 2018;20(9):1138–1143. 29059338 10.1093/ntr/ntx179

[17] Bangladesh Bureau of Statistics, National Tobacco Control Cell, Health Services Division, Ministry of Health and Family Welfare. Global adult tobacco survey 2017 - Bangladesh. 2021; https://extranet.who.int/ncdsmicrodata/index.php/catalog/870 (Accessed April 26, 2022).

[18] Chowdhury SR , Sunna TC, Das DC, et al. Compliance with smoke-free legislation in public places: an observational study in a northeast city of Bangladesh. PLoS One. 2023;18(4):e0283650. 37099518 10.1371/journal.pone.0283650 PMC10132694

[19] Khan JA , Amir Humza Sohail AM, Arif Maan MA. Tobacco control laws in Pakistan and their implementation: A pilot study in Karachi. J Pak Med Assoc. 2016;66(7):875–879. 27427139

[20] Ferdous T , Siddiqi K, Semple S, et al. Smoking behaviours and indoor air quality: a comparative analysis of smoking-permitted versus smoke-free homes in Dhaka, Bangladesh. Tob Control. 2022;31(3):444–451. 33328266 10.1136/tobaccocontrol-2020-055969

[21] Huque R , Siddiqi K, Khokhar M, et al. Children Learning About Secondhand Smoke (CLASS III): a protocol for a cluster randomised controlled trial of a school-based smoke-free intervention in Bangladesh and Pakistan. BMJ Open 2023;13(7):e068620. 10.1136/bmjopen-2022-068620 PMC10351234 37451725

[22] Beal SJ , Dorn LD, Berga SL. Examining the validity of self-reported primary and secondary exposure to cigarette smoke in adolescent girls: the utility of salivary cotinine as a biomarker. Subst Use Misuse. 2018;53(5):792–799. 29058521 10.1080/10826084.2017.1365904 PMC6087668

[23] Liu Q , Shepherd BE, Li C, Harrell FE, Jr. Modeling continuous response variables using ordinal regression. Stat Med. 2017;36(27):4316–4335. 28872693 10.1002/sim.7433 PMC5675816

[24] StataCorp LLC. Stata Longitudinal-Data/Panel-Data Reference Manual Release 18. 2023. http://83.136.219.140:8080/handle/123456789/1677 (Accessed January 20, 2024).

[25] Ripley BD. The R project in statistical computing. MSOR Connections. 2001;1(1):23–25.

[26] rms: Regression Modeling Strategies. Comprehensive R Archive Network (CRAN). https://cran.r-project.org/web/packages/rms/index.html (Accessed January 20, 2024).

[27] Organization WH , Others. Global Adult Tobacco Survey (GATS): Bangladesh Factsheet 2009. 2009. https://extranet.who.int/ncdsmicrodata/index.php/catalog/259/related-materials

[28] Mbulo L , Palipudi KM, Andes L, et al; GATS Collaborative Group. Secondhand smoke exposure at home among one billion children in 21 countries: findings from the Global Adult Tobacco Survey (GATS). Tob Control. 2016;25(e2):e95–e100. 26869598 10.1136/tobaccocontrol-2015-052693 PMC5488799

[29] Farkas AJ , Gilpin EA, White MM, Pierce JP. Association between household and workplace smoking restrictions and adolescent smoking. JAMA. 2000;284(6):717–722. 10927780 10.1001/jama.284.6.717

[30] Morgan C , Parascandola M, Siddiqi K. Secondhand smoke exposure during pregnancy: a cross-sectional analysis of data from Demographic and Health Survey from 30 low-income and middle-income. Tob Contol. 2019;28(4):420–426. https://tobaccocontrol.bmj.com/content/28/4/420.abstract?casa_token=nkv2mUcbaFgAAAAA:otyHAO-VaHCyIyUq_p3sQCoXaDgSCwY_KFGU1oVzSn3uV5miCKHk6zqYxVc1cZu0m99eNiljBxPg 10.1136/tobaccocontrol-2018-054288 PMC10442074 30026189

[31] Zafar Ullah AN , Huque R, Akter S, et al. Children’s exposure to second-hand smoke at home in Bangladesh: a community survey. BMJ Open. 2013;3(11):e003059. 10.1136/bmjopen-2013-003059 PMC3831095 24227868

[32] Abidin EZ , Semple S, Omar A, et al. A survey of schoolchildren’s exposure to secondhand smoke in Malaysia. BMC Public Health. 2011;11(1):634. 21824403 10.1186/1471-2458-11-634 PMC3162528

[33] Martinez-Donate AP , Johnson-Kozlow M, Hovell MF, Gonzalez Perez GJ. Home smoking bans and secondhand smoke exposure in Mexico and the US. Prev Med. 2009;48(3):207–212. 19150456 10.1016/j.ypmed.2008.12.011

[34] Phetphum C , Noosorn N. Prevalence of secondhand smoke exposure at home and associated factors among middle school students in Northern Thailand. Tob Induc Dis. 2020;18(1):11. 32165877 10.18332/tid/117733 PMC7057047

[35] Wakefield M , Banham D, Martin J, et al. Restrictions on smoking at home and urinary cotinine levels among children with asthma. Am J Prev Med. 2000;19(3):188–192. 11020596 10.1016/s0749-3797(00)00197-5

[36] Spencer N , Blackburn C, Bonas S, Coe C, Dolan A. Parent reported home smoking bans and toddler (18-30 month) smoke exposure: a cross-sectional survey. Arch Dis Child. 2005;90(7):670–674. 15970606 10.1136/adc.2004.054684 PMC1720498

[37] Protano C , Andreoli R, Manini P, Vitali M. How home-smoking habits affect children: a cross-sectional study using urinary cotinine measurement in Italy. Int J Public Health. 2012;57(1):885–892. 22434216 10.1007/s00038-012-0354-0

[38] Lindsay RP , Tsoh JY, Sung H-Y, Max W. Secondhand smoke exposure and serum cotinine levels among current smokers in the USA. Tob Control. 2016;25(2):224–231. 25398561 10.1136/tobaccocontrol-2014-051782 PMC8892921

[39] Fallavollita WL , Do EK, Schechter JC, et al. Smoke-Free Home Rules and Association with Child Secondhand Smoke Exposure among Mother–Child Dyad Relationships. Int J Environ Res Public Health. 2021;18(10):5256. 34069235 10.3390/ijerph18105256 PMC8157188

[40] Rosen LJ , Myers V, Winickoff JP, Kott J. Effectiveness of interventions to reduce tobacco smoke pollution in homes: a systematic review and meta-analysis. Int J Environ Res Public Health. 2015;12(12):16043–16059. 26694440 10.3390/ijerph121215038 PMC4690974

[41] Matt GE , Quintana PJE, Hovell MF, et al. Households contaminated by environmental tobacco smoke: sources of infant exposures. Tob Control. 2004;13(1):29–37. 14985592 10.1136/tc.2003.003889 PMC1747815

[42] Akhtar PC , Haw SJ, Currie DB, Zachary R, Currie CE. Smoking restrictions in the home and secondhand smoke exposure among primary schoolchildren before and after introduction of the Scottish smoke-free legislation. Tob Control. 2009;18(5):409–415. 19671536 10.1136/tc.2009.030627

[43] Jarvis MJ , Mindell J, Gilmore A, Feyerabend C, West R. Smoke-free homes in England: prevalence, trends and validation by cotinine in children. Tob Control. 2009;18(6):491–495. 19748885 10.1136/tc.2009.031328

[44] WHO. WHO Report on the Global Tobacco Epidemic, 2011 - The MPOWER package. Geneva: WHO; 2011.

