# Supplementary material for: The Effect of Adult Smoking Behavior on Children’s Exposure to Secondhand Smoke. An Analysis Based on Salivary Cotinine Levels Among Children in Dhaka and Karachi
Source: Nicotine Tob Res. 2024 Jun 19;26(11):1512–20. doi: 10.1093/ntr/ntae130 (PMC11494616; doi:10.1093/ntr/ntae130)

**Appendix 1: Probability of measurements that are BLQ by type of home and country**

**Appendix Figure 1:** Estimated probability of having measurements that are BLQ by type of home (NSH, SFH or SPH), conditional on age (9-15 years), country (Bangladesh or Pakistan), sex (Male or Female), outside space reported at home and SHS exposure reported outside of the home. The point estimates are shown as the darker line with the shaded areas showing pointwise 95% confidence intervals. Note the variation in the labelling of the Y-axis across the four plots.


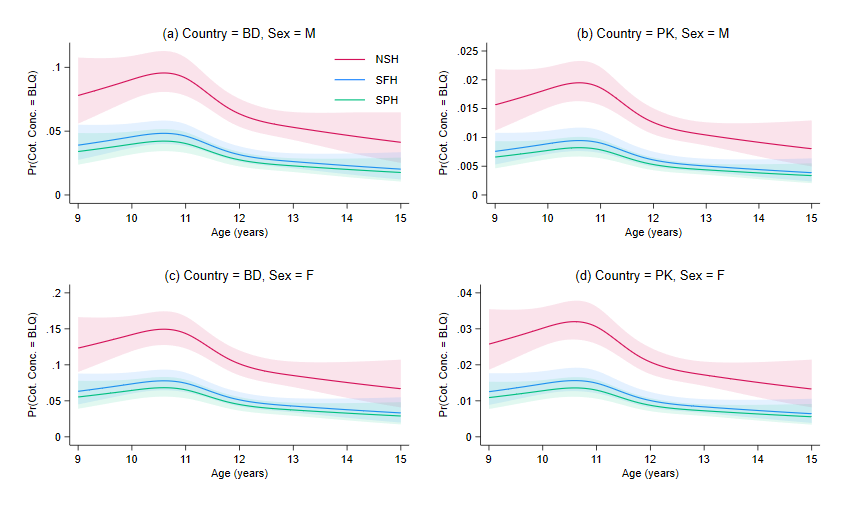


**Appendix Figure 2:** Estimated probability of having measurements that are BLQ by country (Bangladesh or Pakistan), conditional on age (9-15 years), type of home (NSH, SFH or SPH), sex (Male or Female), outside space reported at home and SHS exposure reported outside of the home. The point estimates are shown as the darker line with the shaded areas showing pointwise 95% confidence intervals. Note the variation in the labelling of the Y-axis across the four plots.


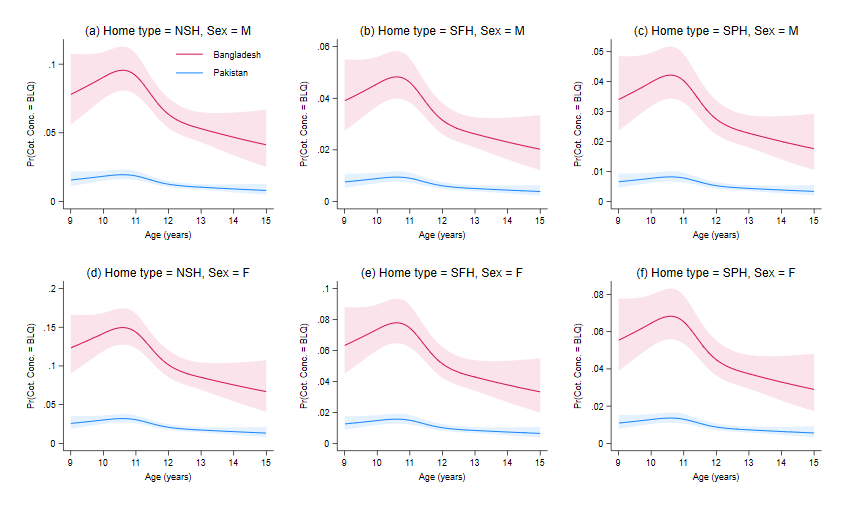

Supplement: ntae130_suppl_Supplementary_Appendix [file ntae130_suppl_supplementary_appendix.docx]
